# The Lectin Complement Pathway Is Involved in Protection Against Enteroaggregative *Escherichia coli* Infection

**DOI:** 10.3389/fimmu.2018.01153

**Published:** 2018-05-29

**Authors:** Camilla Adler Sørensen, Anne Rosbjerg, Betina Hebbelstrup Jensen, Karen Angeliki Krogfelt, Peter Garred

**Affiliations:** ^1^Laboratory of Molecular Medicine, Department of Clinical Immunology, Section 7631, Rigshospitalet, University Hospital of Copenhagen, Copenhagen, Denmark; ^2^Department of Bacteria, Parasites and Fungi, Statens Serum Institut, Copenhagen, Denmark

**Keywords:** enteroaggregative *Escherichia coli*, complement, lectin pathway, ficolin-2, serum resistance

## Abstract

Enteroaggregative *Escherichia coli* (EAEC) causes acute and persistent diarrhea worldwide. Still, the involvement of host factors in EAEC infections is unresolved. Binding of recognition molecules from the lectin pathway of complement to EAEC strains have been observed, but the importance is not known. Our aim was to uncover the involvement of these molecules in innate complement dependent immune protection toward EAEC. Binding of mannose-binding lectin, ficolin-1, -2, and -3 to four prototypic EAEC strains, and ficolin-2 binding to 56 clinical EAEC isolates were screened by a consumption-based ELISA method. Flow cytometry was used to determine deposition of C4b, C3b, and the bactericidal C5b-9 membrane attack complex (MAC) on the bacteria in combination with different complement inhibitors. In addition, the direct serum bactericidal effect was assessed. Screening of the prototypic EAEC strains revealed that ficolin-2 was the major binder among the lectin pathway recognition molecules. However, among the clinical EAEC isolates only a restricted number (*n* = 5) of the isolates bound ficolin-2. Using the ficolin-2 binding isolate C322-17 as a model, we found that incubation with normal human serum led to deposition of C4b, C3b, and to MAC formation. No inhibition of complement deposition was observed when a C1q inhibitor was added, while partial inhibition was observed when ficolin-2 or factor D inhibitors were used separately. Combining the inhibitors against ficolin-2 and factor D led to virtually complete inhibition of complement deposition and protection against direct bacterial killing. These results demonstrate that ficolin-2 may play an important role in innate immune protection against EAEC when an appropriate ligand is exposed, but many EAEC strains evade lectin pathway recognition and may, therefore, circumvent this strategy of innate host immune protection.

## Introduction

Enteroaggregative *Escherichia coli* (EAEC) belongs to the group of diarrheagenic *E. coli* and is an increasingly recognized important cause of diarrhea. EAEC is known to cause watery and often persistent diarrhea in adults as well as children in both industrialized and developing countries. Though several virulence factors are reported, great heterogeneity among EAEC strains has made their molecular epidemiology unclear (1–3).

Enteroaggregative *Escherichia coli* infection is initiated by colonization of the small and large bowel mucosal surfaces by aggregative adherence. This is followed by biofilm formation, induction of an inflammatory response, and release of toxins (1). The precise mechanisms of pathogenesis are still not fully understood, but a combination of several factors such as adhesins and toxins are described to contribute to disease (4, 5). However, none of these factors are conserved in all EAEC strains and a number of similar factors are found in other *E. coli* pathotypes, suggesting that EAEC pathogenesis does not depend on one particular protein, but is probably based on a combination of several virulence factors (2, 4).

Enteroaggregative *Escherichia coli* strains can be recovered from stool samples of apparently healthy individuals and despite studies finding strains associated with diarrhea, some studies have failed to show significant association between EAEC and disease (6–8). This suggests that host factors are involved in manifestations of gastrointestinal disease and further investigations could be crucial for the understanding of EAEC pathogenesis.

The complement system is a complex surveillance system involved in innate immune protection against pathogens. It facilitates opsonophagocytosis of pathogens, induces inflammatory responses, and can lead to bacterial lysis upon activation. Activation can occur *via* three pathways: the lectin, the classical, and the alternative pathway. The complement system is primarily regarded to be of importance for systemic immune protection. But, also local production of complement components is recognized as being important as exudation of complement from the circulation during inflammation appears to be important for local innate immune protection (9).

In the lectin pathway, mannose-binding lectin (MBL) and ficolin-1, -2 and -3 are pattern-recognition molecules (PRMs) involved in initiation of complement activation (10). Recently, two other molecules collectin-10 (CL-10 or CL-L1) and collectin-11 (CL-11 or CL-K1) have to some degree been shown to mediate complement activation (11, 12). They interact with pathogen-associated molecular patterns on the surface of microbial pathogens and upon recognition activate the lectin pathway with help from lectin pathway-associated serine proteases termed MASPs (13). The MASPs cleave C4 and C2 leading to the formation of the C3 convertase (C4b2a). The C3 convertase cleaves C3 into anaphylatoxin C3a and the strong opsonizing factor C3b. Activation through the classical pathway depends on antibody–antigen recognition, which then binds the PRM C1q and leads to cleavage of C4 and C2 by associated proteases C1r/C1s and to deposition of C3b. The alternative pathway is activated spontaneously by hydrolysis of C3, this allows binding of the factor B, which is then cleaved by factor D, forming the C3 convertase of the alternative pathway (C3bBb). The alternative pathway works like an amplification loop for C3b formation and as C3b level rises the C5 convertase is formed (C4b2aC3b/C3bBb3b) initiating formation of the terminal lytic C5b-9 membrane attack complex (MAC) (14).

The involvement of complement in EAEC pathogenesis is unresolved, and though it has previously been shown that ficolin-2 was able to recognize EAEC (15) the importance of the lectin pathway is yet unknown. Thus, we hypothesized that the lectin pathway molecules MBL, ficolin-1, -2, and -3 could be involved in recognition and thus complement dependent protection of EAEC bacteria.

## Materials and Methods

### Bacterial Strains

Four prototype EAEC strains, producing aggregative adherence fimbriae (AAF) I–IV, were investigated for binding of lectin pathway recognition molecules MBL, ficolin-1, ficolin-2, and ficolin-3. The strains have been described previously (16). In addition, 56 EAEC strains isolated from stool samples of Danish adults suffering from diarrhea, at the diagnostic laboratory at Statens Serum Institut, were randomly selected. Stock cultures were frozen at −80°C in Luria-Bertani broth (LB, Sigma-Aldrich) containing 10% (vol/vol) glycerol. Bacteria were cultivated in Dulbecco’s modified eagle medium containing 4.5 g/l d-Glucose (DMEM-HG, Gibco™) overnight with shaking at 37°C until reaching an optical density (OD_600 nm_) of 1.8, corresponding to a bacterial concentration of approximately 5 × 10^8^ cells/ml.

### Proteins

Expression and purification of recombinant proteins was performed as previously described (17). Briefly, MBL and ficolin-1, -2, and -3 were expressed in CHO-DG44 cells cultivated in RPMI 1640 medium (Sigma-Aldrich) supplemented with 10% fetal calf serum (FCS), 100 U/ml penicillin, 0.1 mg/ml streptomycin, 2 mM l-glutamine, and 200 nM methotrexate.

### Consumption Assay

We screened four prototypic EAEC strains for binding of recombinant proteins, and 56 clinical EAEC isolates for binding of serum ficolin-2. The overnight bacterial cultures were centrifuged at 5,000 × *g* for 5 min and washed three times in phosphate-buffered saline (PBS, 137 mM NaCl, 2.7 mM KCl, 2 mM KH_2_PO_4_, and 8.1 mM Na_2_HPO_4_, pH 7.4). The cell pellet was resuspended in Barbital-Tween buffer (Barb-T, 5 mM barbital sodium, 145 mM NaCl, 2 mM CaCl_2_, 1 mM MgCl_2_, 0.05% Tween 20, pH 7.5) [barbital buffer previously used by Rosbjerg et al. (18); Hummelshøj et al. (19)] and incubated with 10% normal human serum (NHS) pool originating from four healthy donors, for 1 h at 4°C, or in-house recombinant proteins for 2 h at 37°C, end-over-end. Recombinant proteins were used in the following concentrations: recombinant ficolin-1 (rficolin-1) 2 µg/ml, rficolin-2 0.5 µg/ml, rficolin-3 0.5 µg/ml, and recombinant MBL (rMBL) 0.5 µg/ml. After centrifugation (5,000 × *g*, 5 min) the supernatant was transferred to quantification assays (described below) where the level of consumption was evaluated by comparing the amount of remaining protein in the supernatant with a control sample containing no bacteria. Serum ficolin-2 screenings were performed using *N*-Acetyl-d-glucosamine-Agarose (GlcNAc) beads (Sigma-Aldrich) as a positive binding control matrix.

### ELISA—Determination of Unbound Protein Fraction

Maxisorp polystyrene microtiter plates (Thermo Scientific) were coated with 5 µg/ml acetylated bovine serum albumin or 10 µg/ml mannan in PBS, overnight at 4°C. Plates were washed and blocked in Barb-T before adding the supernatants (from the consumption assay) in serial dilutions and incubating overnight at 4°C. Plates were washed in Barb-T, and detection was performed using the following primary monoclonal antibodies (mAb) in a concentration of 2 µg/ml at 20°C: anti-ficolin-1 mAb (HP9039, Hycult biotech), anti-ficolin-2 mAb clone FCN219 (20), anti-ficolin-3 clone FCN334 (21), and HYB-131-11 (Bioporto Diagnostics) for MBL detection. Plates were incubated 2 h at room temperature, shaking. Plates were washed and HRP-conjugated rabbit anti-mouse polyclonal antibody (P0260, Dako) (1:1,500) was added for 45 min at room temperature, shaking. Plates were thoroughly washed with Barb-T and subsequently developed for 20 min with tetramethylbenzidine One (TMB ONE, Kem-En-Tec Diagnostics). The reaction was stopped with 0.2 M sulfuric acid (H_2_SO_4_) and OD was measured at 450 nm.

### Western Blot—Detection of Bound Proteins

The bacterial cell pellets (from the consumption assay) were washed thoroughly in Barb-T and analyzed by western blotting. Bacteria were lysed with LDS sample buffer (Invitrogen) and the content was run on a 4–12% bis-Tris polyacrylamide gel (Invitrogen). Rficolin-2 (0.25 µg) was used as a loading control. The separated proteins were blotted onto polyvinylidene difluoride membranes (GE Healthcare) and the membranes were probed with 0.5 µg/ml anti-ficolin-1 mAb FCN106 (cross reacting with ficolin-2) overnight at 4°C (22). After washing, the membranes were incubated with rabbit anti-mouse-HRP (1:10,000) (P0260, Dako) for 1 h at room temperature, shaking. Membranes were developed using SuperSignal West Femto Maximum Sensitivity Substrate (Thermo Scientific).

### Flow Cytometry—Detection of Complement Activation

Prior to assessing binding and activation with flow cytometry, strain C322-17 was fixed in formalin. An overnight culture of C322-17 was washed in PBS and the concentration was determined by a colony-forming unit (CFU) count. Bacteria were fixed in 4% formalin for 40 min, washed in PBS, and resuspended in 50% ethanol. The stock was kept at −80°C until use.

10% NHS diluted in Barbital buffer containing 1% heat-inactivated FCS (Barb-FCS) were combined with the following inhibitors in a concentration of 5 µg/ml: ficolin-2 inhibitory mAb FCN212 (18), anti-factor D inhibitory mAb clone 31A9 (anti-FD, a kind gift from Genentech), C1q inhibitory mAb clone 85 (C1q-85, Sanquin), and mock inhibitor mouse IgG1 (BD Biosciences). Besides, ethylene diamine tetraacetic acid (EDTA) 10 mM (324503, Calbiochem, Merck) was applied. 5 × 10^7^ fixed cells/ml were added for 30 min at 37°C. Bacteria were washed in Barb-FCS and spun down at 5,000 × *g* for 5 min at 4°C. Deposition of C4b, C3b, and C5b-9 were measured using biotinylated anti-C4c pAb (A0065, Dako), biotinylated anti-C3c pAb (A0062, Dako) and biotinylated anti-MAC mAb clone aE11 (23). Anti-ficolin-2 mAb FCN219 (20) was used to detect ficolin-2. Primary antibodies were applied in a concentration of 5 µg/ml for 30 min at 4°C. The specificity of the primary antibodies were verified using a biotinylated rabbit IgG (Rb IgG, 10500C, Invitrogen) or biotinylated mouse IgG2 (mIgG2a, 553455, BD Pharmingen) isotype control. After washing, FITC-conjugated streptavidin (Strep-FITC, S3762, Sigma-Aldrich) were added for 30 min at 4°C. The samples were washed and the levels of bacterial-bound C3, C4, and MAC were measured by flow cytometry using Gallios Flow Cytometer (Beckman-Coulter) and data were analyzed using Kaluza 1.2 software (Beckman-Coulter).

### Microscopy

Ficolin-2 binding in the presence and absence of the ficolin-2 inhibitor FCN212 was assessed by microscopy. The residual EAEC cells from flow cytometry was placed on slides by cytospin (centrifugation for 5 min at 300 × *g*) and mounted with ProLong Diamond Antifade Mountant (P36965, Life Technologies). Microscopy was performed using a Zeiss Axio Observer through a X63/1.40 oil DIC Plan-Apochromat objective. Imaging conditions were kept constant when acquiring images to be compared.

### EAEC Serum Resistance

An overnight bacterial culture of C322-17 was diluted to an OD = 0.5 at 600 nm in PBS. 10% NHS were incubated in buffer containing the following inhibitors: FCN212 (5 µg/ml), anti-FD (5 µg/ml), C1q-85 (5 µg/ml) or mock inhibitor mAb Ciona (a mAb raised against an MBL homolog in *Ciona intestinalis*). In addition, we used a specific peptide inhibitor of C3 activation, compstatin (CP40, 6 µM, a kind gift from professor John Lambris, Philadelphia, PA, USA), and C5 inhibitor eculizumab (50 µg/ml, Soliris, Alexion Pharmaceuticals). Heat-inactivated NHS (IHS) (56°C, 30 min) was included as a negative control of complement activation. The baseline consisted of bacteria incubated only with buffer to assess the number of viable cells in the initial inoculum.

All tubes were preincubated at 37°C for 30 min to let the inhibitors work and to start the test approximately at human blood temperature. Then, 20 µl of the inoculum was added to each tube and the sample was homogenized. The samples were incubated at 37°C and 20 µl was spotted in a serial dilution of 10^−1^ to 10^−6^ on LB agar plates and incubated at 37°C overnight before CFU was assessed. The control sample with viable cells of the initial inoculum was diluted and plated at 0 min incubation.

### Statistics

Statistical analyses were performed using GraphPad Prism 7 (GraphPad Software, USA). The results represent the means ± SD of three independent experiments. For comparisons, we used an unpaired Student’s *t*-test (*p-*values: ns *p* > 0.05; *p ≤ 0.05; **p ≤ 0.01; ***p ≤ 0.001; ****p ≤ 0.0001).

## Results

### Binding of rMBL and Ficolins to Prototypic EAEC Strains

**Figure 1:**
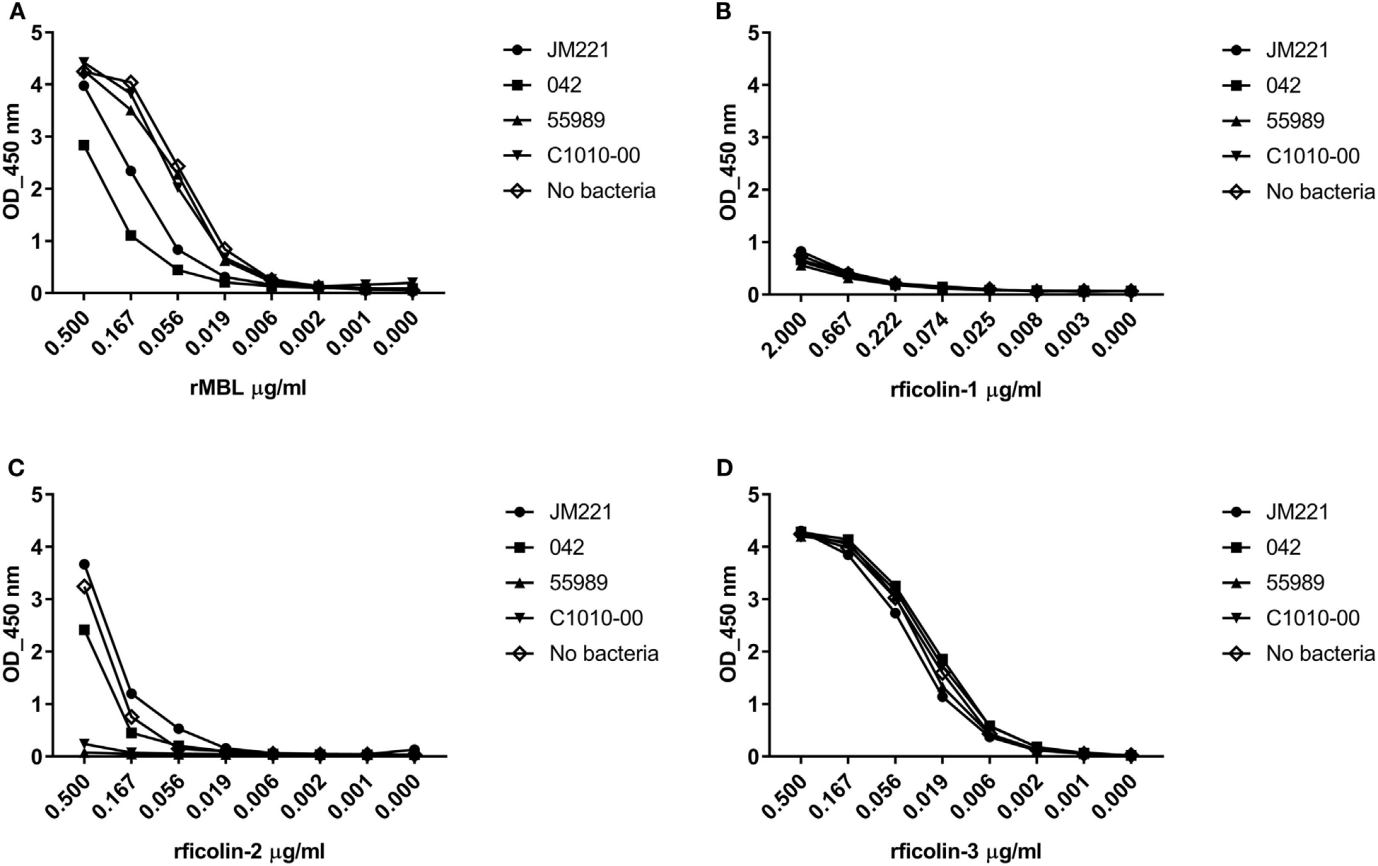
Consumption of recombinant MBL (rMBL), rficolin-1, -2, and -3 by prototypic EAEC strains. EAEC prototype strains JM221, 042, C1010-00, and 55989 were incubated with **(A)** rMBL (0.5 µg/ml), **(B)** rficolin-1 (2.0 µg/ml), **(C)** rficolin-2 (0.5 µg/ml), or **(D)** rficolin-3 (0.5 µg/ml) and the unbound protein fraction was transferred to wells coated with mannan or acetylated bovine serum albumin and detected by specific mAb against mannose-binding lectin (MBL), ficolin-1, -2, or -3.

Four prototypic EAEC strains were screened in a consumption assay for binding of rMBL, rficolin-1, -2, and -3. Neither rficolin-1 nor rficolin-3 appeared to bind to any of the strains (Figures 1B,D), whereas rMBL showed binding to two of the strains (JM221 and 042) (Figure 1A). The strongest binding, however, was observed for rficolin-2, which displayed high binding to two strains (55989 and C1010-00) and to a lesser degree to strain 042 (Figure 1C). We confirmed the binding by performing a Western blot on the eluates from the consumption assay (data not shown).

We found that rficolin-2 displayed highest binding to the four prototypic EAEC strains and, therefore, decided to examine ficolin-2 binding to 56 clinical EAEC isolates.

### Binding of Serum Ficolin-2 to 56 Clinical EAEC Isolates

**Figure 2:**
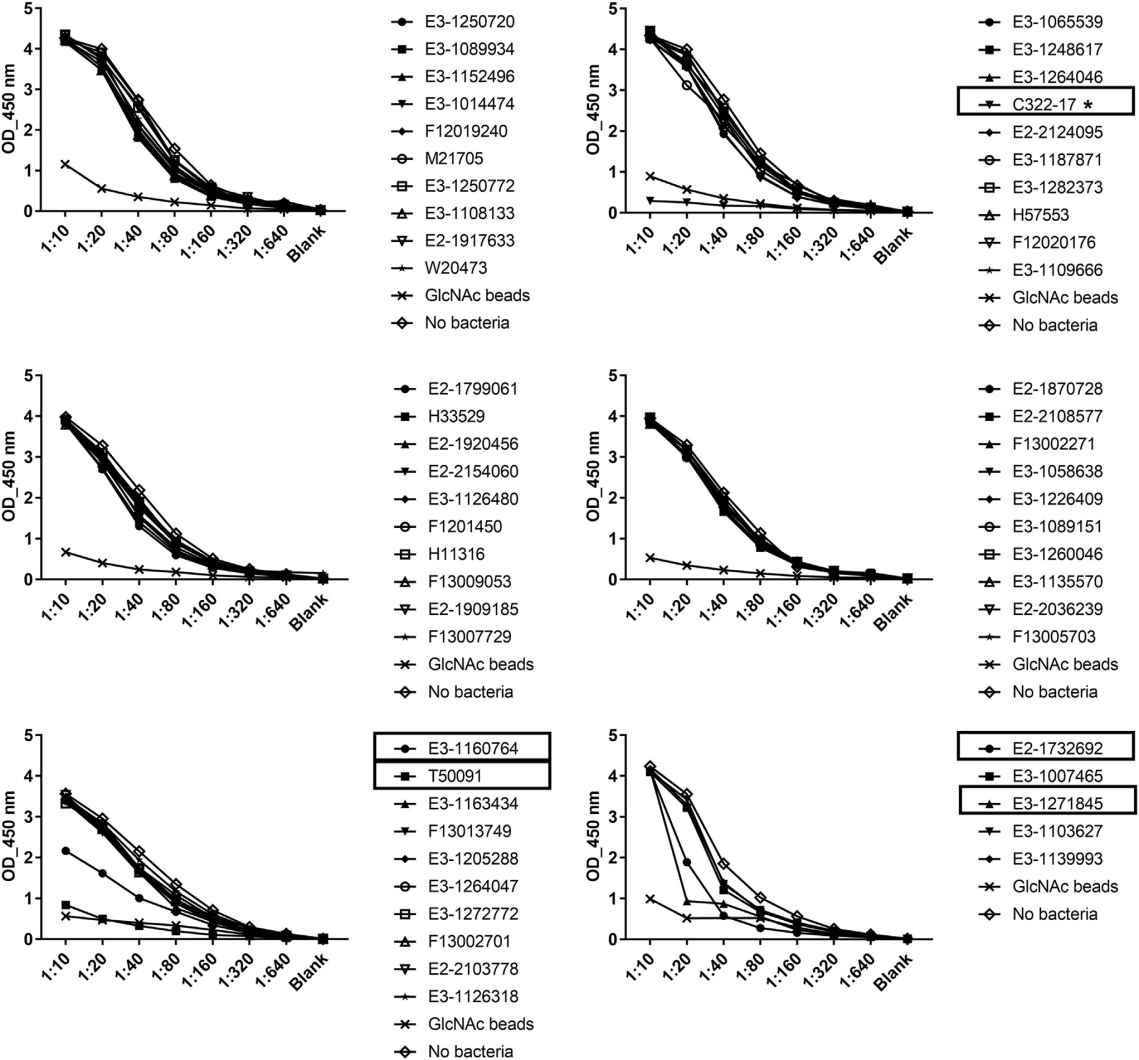
Consumption of serum ficolin-2 by 56 clinical Enteroaggregative *Escherichia coli* (EAEC) isolates. 56 clinical EAEC isolates and a control containing GlcNAc beads were incubated with 10% normal human serum and the unbound protein fraction was transferred to ELISA plates coated with acetylated bovine serum albumin, in twofold serial dilutions. Specific mAb were used to detect ficolin-2. Boxes show isolates binding ficolin-2 and * marks isolate C322-17 showing high binding of ficolin-2.

**Figure 3:**
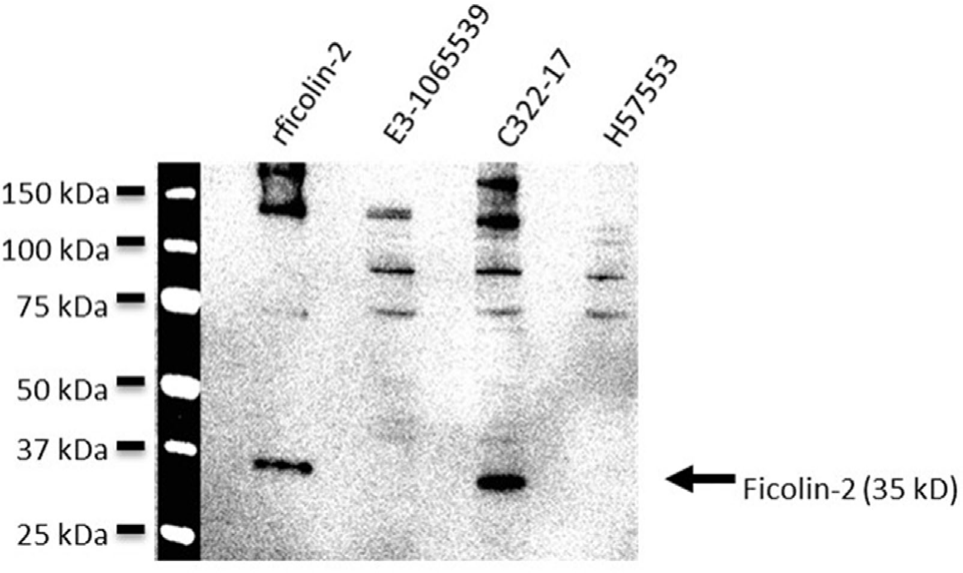
Western blot detection of bound serum ficolin-2 to isolate C322-17. Isolates C322-17 (high binding), E3-1065539 (low binding), and H57553 (low binding) was incubated with 10% normal human serum and the eluates were separated by SDS PAGE and detected by western blot. 0.25 µg rficolin-2 was added as a positive control and specific mAb was used to detect ficolin-2.

56 clinical EAEC isolates obtained from adult patients suffering from EAEC-related diarrhea were screened for binding of serum ficolin-2 and the unbound fraction of serum ficolin-2 was measured. Five (8.9%) of the 56 isolates appeared to bind serum ficolin-2 and especially one isolate, C322-17, showed very strong binding (Figure 2). We furthermore verified that serum ficolin-2 was bound to isolate C322-17 by performing a Western blot on the bacterial pellet for C322-17 and two isolates that were negative for ficolin-2 binding according to the ELISA. The Western blot showed strong monomeric and oligomeric ficolin-2 structures to be associated with C322-17. The two isolates, E3-1065539 and H57553, which were negative for ficolin-2 binding in the ELISA displayed low levels of oligomeric ficolin-2 binding, but no monomeric bands were observed (Figure 3).

Since isolate C322-17 displayed high binding of ficolin-2, we decided to use this isolate as a model for further investigation of EAEC-associated complement binding and activation.

### Calcium Dependency of Ficolin-2 Binding

**Figure 4:**
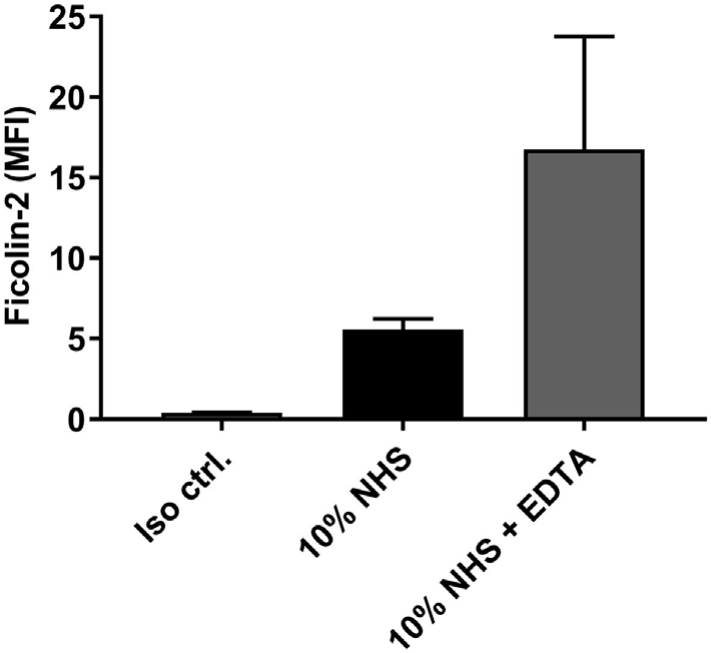
Ficolin-2 binding is independent of calcium. Isolate C322-17 was incubated with 10% normal human serum (NHS) in Barb-fetal calf serum (FCS), and 10% NHS in Barb-FCS + 10 mM ethylene diamine tetraacetic acid (EDTA). Binding of ficolin-2 was detected with specific biotinylated mAb against ficolin-2 and FITC-conjugated Streptavidin. Complement deposition was measured by flow cytometry and expressed as mean fluorescence intensity (MFI). Results represent the means of three independent experiments ± SD.

We examined whether ficolin-2 binding to isolate C322-17 was calcium dependent by pre-incubating NHS with 10 mM EDTA. Figure 4 shows the mean fluorescence intensities (MFI) when detecting ficolin-2 binding in flow cytometry. Ficolin-2 binding was not reduced by EDTA providing evidence that that the binding was calcium independent. In fact, it seemed that EDTA enhanced binding of ficolin-2, perhaps due to elimination of other calcium-dependent binding partners.

### Complement Activation by Ficolin-2 Through the Lectin Pathway

**Figure 5:**
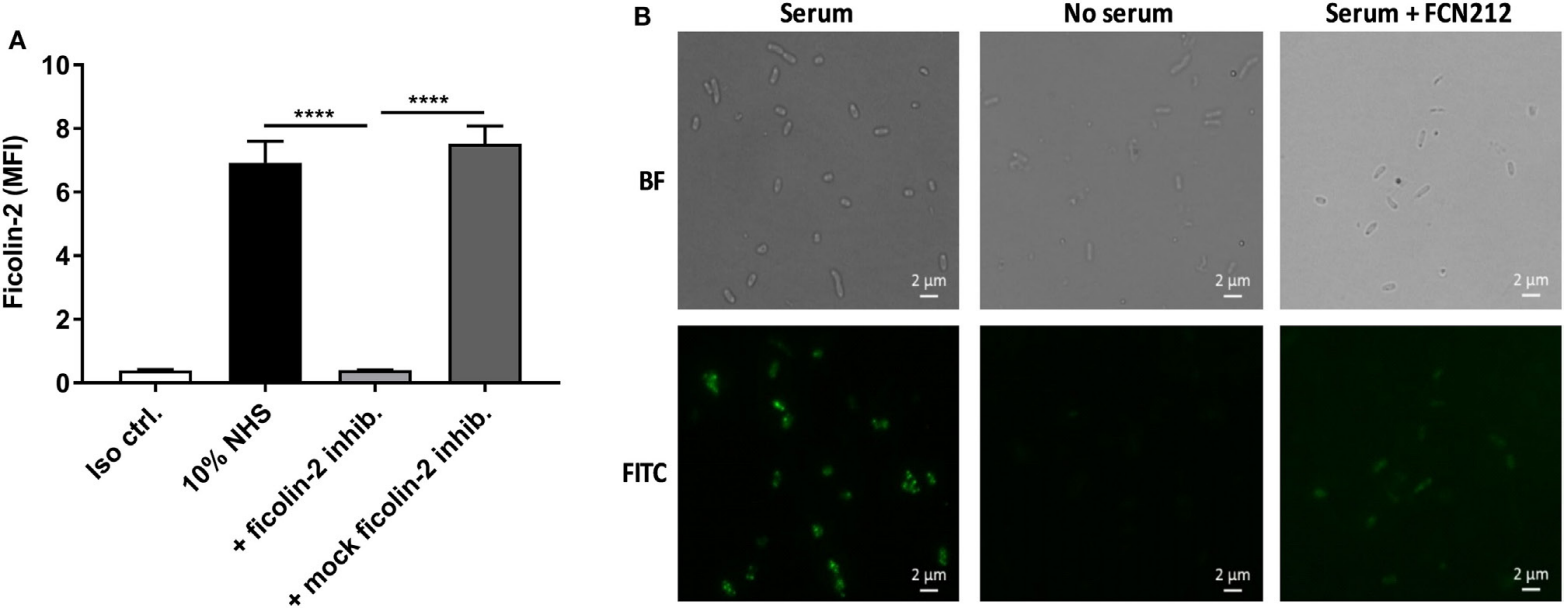
Inhibitory effects of ficolin-2 inhibitor on ficolin-2 binding. 10% normal human serum (NHS) was combined with ficolin-2 inhibitor FCN212 (5 µg/ml) and incubated with isolate C322-17. Detection was performed with specific biotinylated monoclonal antibodies against ficolin-2, followed by FITC-conjugated Streptavidin. **(A)** Ficolin-2 deposition was measured by flow cytometry and expressed as mean fluorescence intensity (MFI). **(B)** Residual EAEC cells from flow cytometry was placed on slides by cytospin and examined by bright field (BF) and fluorescent microscopy (FITC). Results represent the means of three independent experiments ± SD, ****p ≤ 0.0001 (unpaired Student’s *t*-test).

The contribution of ficolin-2 to lectin pathway complement activation was assessed by introducing FCN212, a ficolin-2 inhibitory mAb. Figures 5A,B shows inhibition of ficolin-2 binding to the bacterial strain C322-17 in flow cytometry and fluorescence microscopy, respectively, when applying the inhibitor. Both techniques showed that the binding was reduced by the employed ficolin-2 inhibitor. In flow cytometry, the inhibition was significantly reduced both when comparing to 10% NHS and to a mock ficolin-2 inhibitor (*p* < 0.0001).

**Figure 6:**
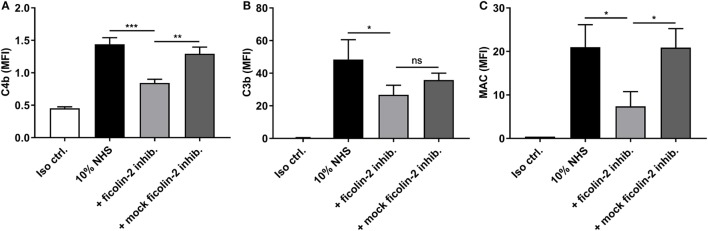
Inhibitory effects of a ficolin-2 inhibitor on C4b, C3b, and membrane attack complex (MAC) deposition. 10% normal human serum (NHS) was combined with ficolin-2 inhibitor FCN212 (5 µg/ml) and incubated with isolate C322-17. Detection was performed with specific biotinylated monoclonal antibodies against **(A)** C4b, **(B)** C3b, and **(C)** MAC, followed by FITC-conjugated Streptavidin. Complement deposition was measured by flow cytometry and expressed as mean fluorescence intensity (MFI). Results represent the means of three independent experiments ± SD, ns *p* > 0.05, *p ≤ 0.05, **p ≤ 0.01, ***p ≤ 0.001 (unpaired Student’s *t*-test).

The effect of ficolin-2 binding on complement activation was examined by looking at the deposition of C4b, C3b, and MAC in the presence and absence of the ficolin-2 inhibitor. C4b deposition was significantly reduced by the inhibitor when comparing to both 10% NHS and the mock inhibitor (*p* = 0.0008 and 0.0026, respectively). The same tendencies were observed for MAC deposition when comparing to 10% NHS and the mock inhibitor (*p* = 0.0189 and 0.0134, respectively), suggesting an involvement of ficolin-2 and the lectin pathway in complement activation (Figures 6A,C). We observed a small significant reduction in C3b deposition when comparing to 10% NHS (*p* = 0.0495), but were unable to detect a significant reduction when comparing to the mock inhibitor (*p* = 0.0964), but overall the tendency followed that of C4b and MAC (Figure 6B). We also looked at MBL binding to see if this was involved in activation *via* the lectin pathway, but MBL did not bind to C322-17 and thus does not contribute to activation (data not shown).

### The Classical Pathway Does Not Show Involvement in Complement Activation

**Figure 7:**
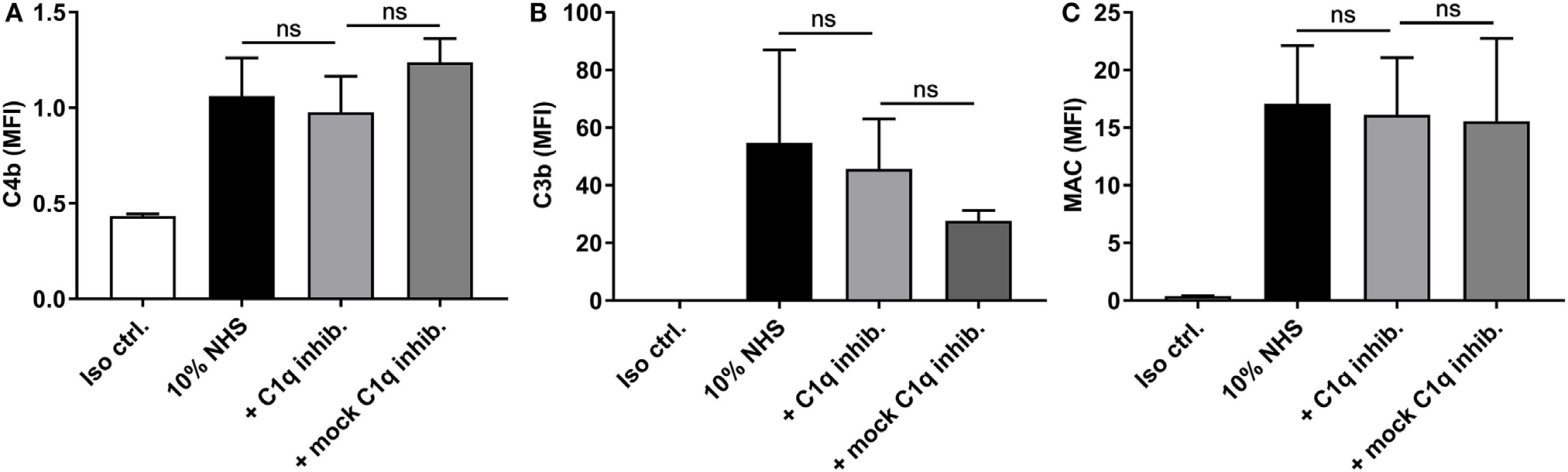
Inhibitory effects of a C1q inhibitor on C4b, C3b, and membrane attack complex (MAC) deposition. 10% normal human serum (NHS) was combined with C1q inhibitor C1q-85 (5 µg/ml) and incubated with isolate C322-17. Detection was done with specific biotinylated monoclonal antibodies against **(A)** C4b, **(B)** C3b, and **(C)** MAC, followed by FITC-conjugated Streptavidin. Complement deposition was measured by flow cytometry and expressed as mean fluorescence intensity (MFI). Results represent the means of three independent experiments ± SD. No significant differences were detected between the C1q inhibitor and the mock inhibitor, ns *p* > 0.05 (unpaired Student’s *t*-test).

Since ficolin-2 only seemed to be partially involved in complement activation, we investigated whether the remaining activity was initiated *via* the classical pathway. When introducing an inhibitor of C1q, we saw no significant reduction in the deposition of C4b, C3b, or MAC (*p* > 0.05) (Figures 7A–C, respectively) and, therefore, the classical pathway does not appear to be involved in the remaining complement activity.

### The Alternative Pathway Contributes to Complement Activation

**Figure 8:**
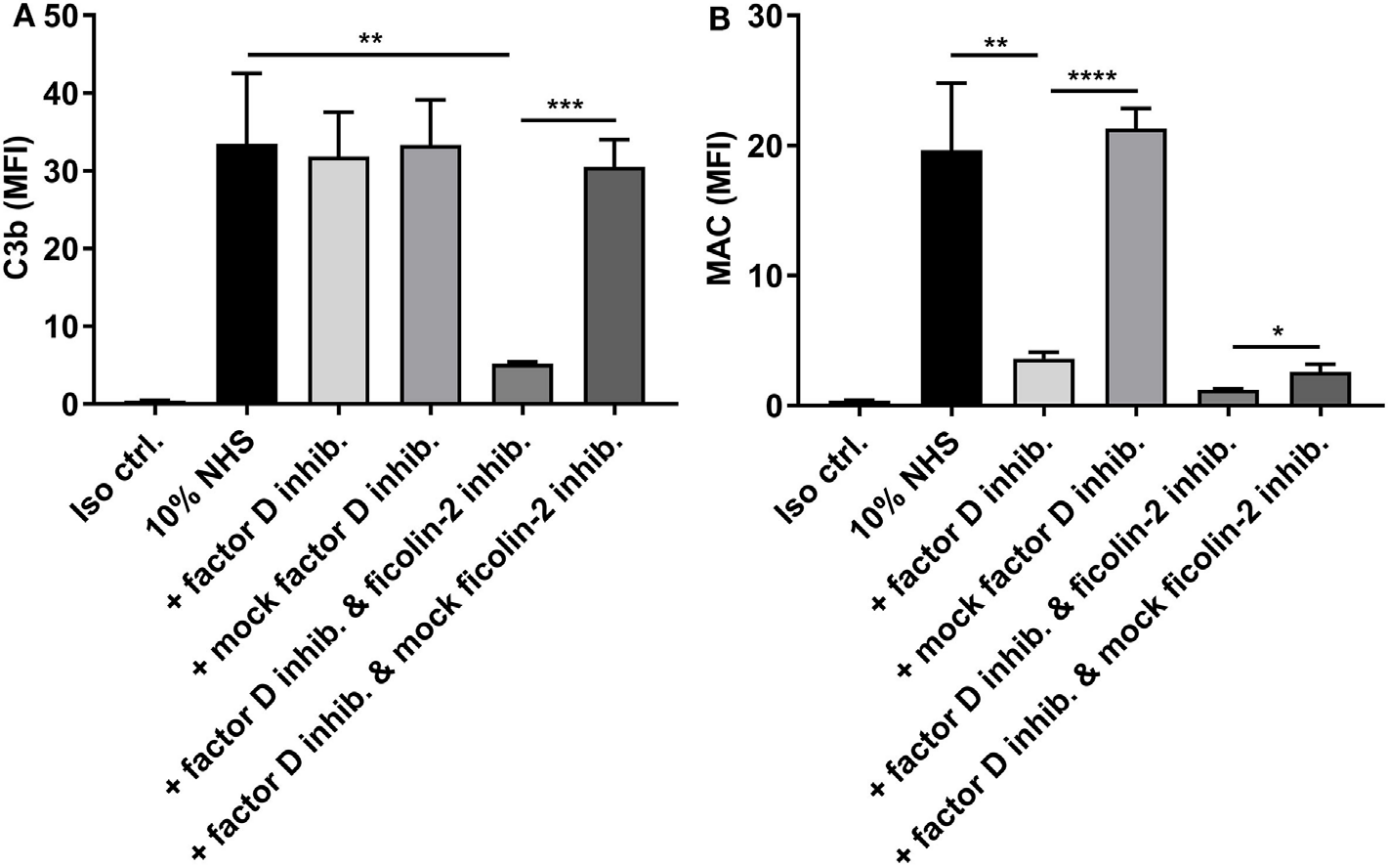
Inhibitory effects of a factor D inhibitor on C3b, and membrane attack complex (MAC) deposition. 10% normal human serum (NHS) was combined with a factor D inhibitor (5 µg/ml) and incubated with isolate C322-17. Detection was done with specific biotinylated monoclonal antibodies against **(A)** C3b and **(B)** MAC, followed by FITC-conjugated Streptavidin. Complement deposition was measured by flow cytometry and expressed as mean fluorescence intensity (MFI). Results represent the means of three independent experiments ± SD, *p ≤ 0.05, **p ≤ 0.01, ***p ≤ 0.001, ****p ≤ 0.0001 (unpaired Student’s *t*-test).

Next, we tested the involvement of the alternative pathway by applying an inhibitory antibody against factor D before detecting C3b and MAC deposition (Figure 8). We were unable to detect a significant reduction in C3b levels when introducing the factor D inhibitor alone. However, when combining the factor D inhibitor with the ficolin-2 inhibitor, we observed a significant reduction in C3b deposition when comparing both to 10% NHS and to a sample where the ficolin-2 inhibitor was exchanged with a mock inhibitor (*p* = 0.0057 and 0.0002, respectively) (Figure 8A). The deposition of MAC was highly reduced by the factor D inhibitor alone (10% NHS *p* = 0.0058 and mock inhibitor *p* < 0.0001) and adding the ficolin-2 inhibitor led to further reductions when comparing to the mock inhibitor (*p* = 0.0169) (Figure 8B).

These data suggest that both the lectin pathway and the alternative pathway are important in activation of complement on EAEC strain C322-17 and emphasize the importance of the alternative pathway in generation of MAC.

### Ficolin-2 and Factor D Are Involved in Serum-Mediated EAEC Killing

**Figure 9:**
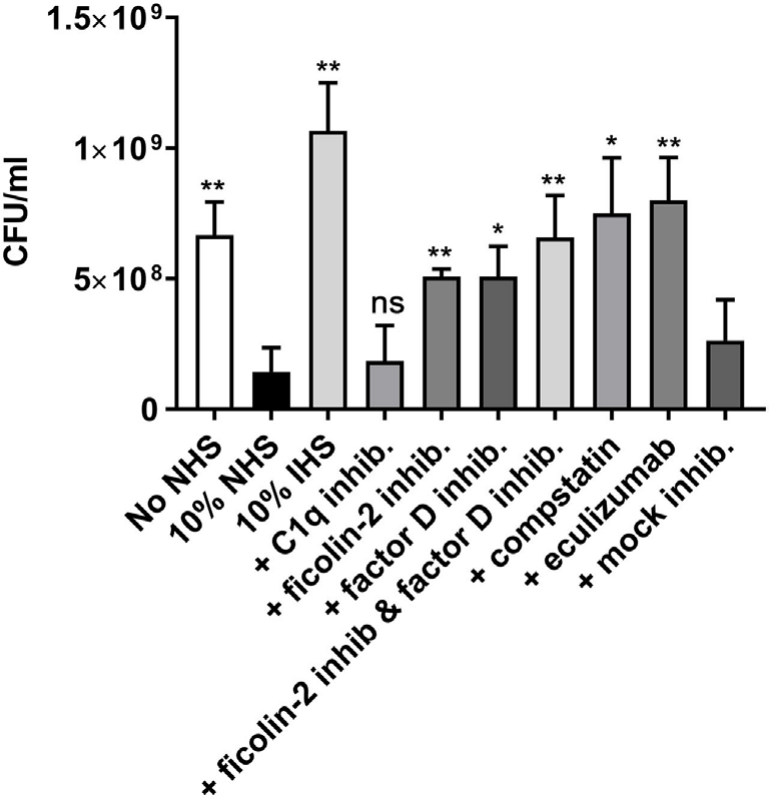
Inhibitors of ficolin-2 and factor D reduce serum-mediated enteroaggregative *Escherichia coli* (EAEC) killing. Bacterial growth in normal human serum (NHS) measured by colony-forming unit (CFU)/ml. EAEC isolate C322-17 was incubated with 10% NHS in combination with complement inhibitors of ficolin-2, factor D, C1q, C3 (compstatin), and C5 (eculizumab). A baseline (No NHS) was established from an overnight culture under normal growth conditions without the presence of NHS or inhibitors. Results represent the means of three independent experiments ± SD. All conditions are compared to 10% NHS, ns *p* > 0.05, *p ≤ 0.05, **p ≤ 0.01 (unpaired Student’s *t*-test).

To further investigate the involvement of ficolin-2 and the alternative pathway on bacterial clearance, we performed a serum resistance assay where bacteria were mixed with 10% NHS and the inhibitors of ficolin-2, factor D and C1q, as well as the C3-targeted complement inhibitor, compstatin, and the terminal complement inhibitor eculizumab that prevents MAC formation. We assessed the change in CFU/ml between 10% NHS and the different parameters. There was a significant reduction when bacteria were grown in 10% NHS compared to the baseline (No NHS) (*p* = 0.0046). Heat-inactivated human serum (IHS) was applied as a control of complement activity and did not only lead to rescue of bacterial growth, but could be interpreted to function as a growth medium for the bacteria. The C1q inhibitor did not rescue the bacteria (*p* = 0.6904), suggesting no involvement from the classical pathway in the observed serum-mediated killing. The ficolin-2 inhibitor and the factor D inhibitor each led to significant bacterial rescue (*p* = 0.0029 and 0.0130, respectively), suggesting involvement of both the lectin and alternative pathway in serum-mediated killing. Combining the two inhibitors did not appear to further increase the rescue. Targeting C3 and MAC with inhibitors also led to significant rescue of bacterial growth (*p* = 0.0107 and 0.0038, respectively) (Figure 9).

## Discussion

Enteroaggregative *Escherichia coli* (EAEC) is a well-known diarrheagenic pathogen, causing acute and persistent diarrhea in children and adults worldwide. However, the molecular epidemiology of EAEC still remains unclear and several studies have recovered EAEC from stool samples of apparently healthy individuals suggesting the involvement of host factors (2).

The lectin pathway of the complement system relies on PRMs to assist in the clearance of microbial intruders. Ficolins are a family of PRMs belonging to the lectin pathway. They bind structures such as *N*-acetyl-glucosamine (GlcNAc), *N*-acetyl-galactosamine (GalNAc) and acetylated compounds on target cells (24). Although complement proteins are generally considered in the systemic compartment, recent studies show production and secretion of complement components in human immune cells, as well as endothelial and epithelial cells (9). This makes the study of interactions between rare or non-systemic pathogens and complement highly relevant and could potentially give a better understanding of host defense systems, as well as bacterial pathogenesis.

In this study, we assessed the involvement of the lectin complement pathway on 56 EAEC strains isolated from patients suffering from EAEC-related diarrhea. First, we applied a consumption assay to screen the binding of rMBL and ficolins to prototypic EAEC strains in an ELISA setup. We found that rficolin-2 presented with the highest binding capacities for the prototype EAEC strains, showing strong binding to prototype strains 55989 and C1010-00, and to some degree to prototype 042. In a previous study no binding of ficolin-2 to prototype strain *E. coli* 042 was detected, but they were using NHS (serum protein) and in a different *E. coli* growth medium (15). Using a different growth medium for EAEC could potentially change the gene expression of the strain and lead to changes in surface presentation. Biofilm formation in some EAEC strains have shown to increase significantly when using a high glucose containing medium as compared to regular LB (25). This emphasizes the importance of the methodology employed when studying bacterial interaction with host factors.

Based on our initial screenings, we focused on the PRM ficolin-2. Previous reports have described binding of ficolin-2 to Gram-positive bacteria, such as group B *streptococci, S. pneumoniae, S. pyogenes*, and capsulated *S. aureus* (26–28), but very few studies have described binding of ficolin-2 to Gram-negative bacteria. A study by Sahagún-Ruiz et al. explored the binding capacity of serum ficolin-2 and ficolin-3 to Gram-negative bacteria including four EAEC strains. They found that ficolin-2 and ficolin-3 recognized one EAEC strain (serotype O71), but not the other three. By testing binding to another EAEC serotype O71 they concluded that binding was not related to the bacterial LPS type (15), but did not determine the specific binding factor.

The binding capacity of the 56 clinical EAEC isolates was screened by incubating bacteria with 10% NHS. Ficolin-2 binding was only detected in five of the 56 EAEC isolates (8.9%). Complement evasion strategies are numerous and include mimicking or recruitment of complement regulators, inhibition of complement proteins, and enzymatic degradation leading to inactivation (29). Both Gram-positive and Gram-negative bacteria are capable of evading complement by recruitment of complement regulators, such as factor H and C4b-binding protein (C4BP) (30). The *E. coli* K1, causing neonatal meningitis, utilizes the outer membrane protein A (OmpA) as protection against complement-mediated killing, and serum resistance was correlated with the binding of C4bP to OmpA (31, 32). Immune evasion has also been reported for EAEC by cleavage of complement proteins by Pic, which is a serine protease present in approximately 50% of clinical EAEC isolates (4, 5, 33, 34). In a study by Abreu et al., they showed that Pic significantly reduced complement activation by cleavage of C3, C3b, C4, and C2, thereby affecting all three complement pathways (35). EAEC is known to cause persistent diarrhea, most likely due to the formation of a resilient biofilm. A study from 2013 showed that biofilm formation worked as an efficient way of evading complement detection (36) and could potentially be a way for EAEC to avoid complement. As part of the present study, we investigated whether ficolin-2 binding could be related to the five EAEC characteristic AAF (I–V). By PCR, we characterized the presence of AAFs in the 56 clinical EAEC strains, but we were unable to find a link between binding of ficolin-2 and the presence of a specific AAF, the strains that bound ficolin-2 harbored different AAFs (data not shown). We hypothesize that some bacterial strains are able to change their surface composition in order to avoid ficolin-2 binding, thus escaping the initiating effect of complement.

It is important to mention that we did see a difference in detection of ficolin-2 binding when comparing ELISA with western blot. Two of the strains reported negative for binding in the ELISA, were run on a western blot where we were able to detect low levels of oligomeric binding. This should be tested further to determine the sensitivity of the ELISA.

Before assessing the complement activation potential of EAEC, we determined whether ficolin-2 binding was calcium dependent. X-ray crystallographic analysis has revealed four different binding sites in the fibrinogen-like domain of ficolin-2, enabling binding to a wide variety of ligands (24). While some of these binding sites require the presence of calcium, others can bind calcium independently (10). Using isolate C322-17 as a model for high level ficolin-2 binding, we could show that ficolin-2 binding to the bacteria was calcium-independent.

We next showed that ficolin-2 binding to EAEC can lead to activation of complement. The activation could be partially inhibited by a ficolin-2-specific inhibitor. Furthermore, activation was independent of the classical pathway, thereby confirming the involvement of the lectin pathway of complement. We also show that the alternative pathway contributes to activation and that the amplification loop contributes to a high production of C3b, possibly explaining the need to introduce both a ficolin-2 inhibitor and a factor D inhibitor to see a decreasing effect on C3b levels. This observation is compatible with previous studies on fungi showing the importance of alternative pathway amplification on lectin pathway-initiated activation (18).

Finally, we show that ficolin-2 and factor D are involved in serum-mediated killing of EAEC and that this killing is completely dependent on the formation of the MAC complex. Although EAEC is not common in sepsis infections, there have been reports of EAEC-induced bacteremia (37, 38), and this could be due to lack of complement dependent systemic control of the infection.

## Conclusion

This study shows for the first time a crucial influence of ficolin-2 in the control of some strains of EAEC bacteria, but it also shows that only a fraction of the strains indeed binds ficolin-2 by mechanisms that remain to be elucidated. This suggests that many EAEC strains may evade ficolin-2 and probably other innate immune recognition mechanisms in their pathogenic survival strategies.

## Author Contributions

CS: study design, experimental work, data interpretation, drafting the article, and final approval. AR, KK, and PG: study design, data interpretation, critical revision of the article, and final approval. BJ: collection of clinical strains used in the study and final approval.

## Conflict of Interest Statement

The authors declare that the research was conducted in the absence of any commercial or financial relationships that could be constructed as a potential conflict of interest.
